# Prognostic and predictive capacity of tumor infiltrating lymphocytes in the MA.20 regional node radiotherapy trial

**DOI:** 10.1038/s41523-025-00821-z

**Published:** 2025-08-29

**Authors:** N. Riaz, B. E. Chen, A. Bane, D. Gao, E. S. Stovgaard, Z. Kos, S. C. Leung, E. Shenasa, W. Parulekar, S. Chambers, T. O. Nielsen, T. J. Whelan

**Affiliations:** 1 Department of Pathology and Laboratory Medicine, University of British Columbia, Vancouver, BC Canada; 2 Department of Surgery, Institute of Clinical Sciences, Gothenburg University, Gothenburg, Sweden; 3 Canadian Clinical Trials Group, Queen’s University, Kingston, ON Canada; 4 University Health Network, Department of Laboratory Medicine and Pathobiology, University of Toronto, Toronto, ON Canada; 5 Department of Pathology, Herlev and Gentofte Hospital, Copenhagen, Denmark; 6 Department of Clinical Medicine, University of Copenhagen, Copenhagen, Denmark; 7 Department of Oncology, McMaster University, Hamilton, ON Canada; 8 Division of Radiation Oncology, Juravinski Cancer Centre at Hamilton Health Sciences, Hamilton, ON Canada

**Keywords:** Biomarkers, Cancer, Breast cancer, Tumour biomarkers, Tumour immunology

## Abstract

Prognostic and predictive value of immune infiltrates in the context of regional nodal radiation (RNI) for breast cancer has not been assessed. Stromal tumor infiltrating lymphocytes (sTILs) were assessed on scanned images of hematoxylin and eosin (H&E) stained sections and by CD8 immunohistochemistry on tissue microarrays available from the MA.20 trial. Cox proportional modelling was used, and hazard ratios (HR) with 95% confidence intervals (CI) are reported for primary and secondary endpoints. Predictive value was assessed by an interaction test. H&E sTILs (continuous parameter) were prognostic for distant-DFS (HR 0.99, 95% CI 0.98–1.00, *P* = 0.04). CD8+sTILs were associated with significantly improved disease-free survival (DFS) (HR 0.99, 95% CI 0.98–1.00, *P* = 0.02) and distant-DFS (HR 0.98, 95% CI 0.97–0.99, *P* = 0.0002). CD8+sTILs was predictive of benefit from RNI for distant-DFS (continuous variable: HR 0.98, 95% CI 0.96–1.00, *P*_(interaction)_ = 0.04; exploratory categorical variable: CD8+ sTILs < 44, HR = 0.83; 95% CI 0.57–1.21, and CD8+ sTILs ≥ 44; HR 0.09; 95% CI 0.01–0.74, *P*_(interaction)_ = 0.04). In MA.20 breast cancer patients, pre-treatment sTILs were prognostic for DFS (CD8+sTILs) and distant-DFS. CD8+sTILs also appeared to be predictive for the effectiveness of RNI on distant-DFS, suggesting that immune mechanisms may in part be responsible and merits further investigation.

## Introduction

High levels of stromal tumor-infiltrating lymphocytes (sTILs) reflect the robustness of host anti-tumor immunity in most solid organ malignancies, including breast cancer^1^. Assessment of sTILs on hematoxylin and eosin (H&E) stained sections has shown reliability, reproducibility, and feasibility as a validated biomarker for gauging pre-existing anti-tumoral immunity using readily available and inexpensive methodologies. Although the value of TILs in luminal breast cancers and, in particular, the luminal A subtype remains ambiguous^2,3^, high TIL levels have demonstrated strong clinical value in non-luminal subtypes. Particularly, in early-stage triple-negative breast cancer (TNBC) treated with upfront surgery and adjuvant chemotherapy, high baseline TILs have been shown to be prognostic for survival^4^ and predictive of pathological complete response after neoadjuvant chemotherapy^5,6^. Recently, high TILs have also been shown to be prognostic in chemo naïve early-stage TNBC, with potential implications for guiding chemotherapy de-escalation in select low-risk patients^7^. Likewise, in the human epidermal growth factor receptor 2 (HER2) subtype, TIL levels have shown favorable prognostic and predictive value in adjuvant settings, though the value for predicting response to neoadjuvant dual HER2 targeted therapies has been variable^8^. Estimation of TILs is endorsed by clinical practice guidelines with IB level of evidence for prognostication^9^ and supported for assessment as an essential biomarker for the selection of breast cancer patients in immuno-oncology trials^9,10^.

Having demonstrated the capacity to predict response from cytotoxic and immune checkpoint inhibitor therapies^4,11^ and the potential for de-escalating chemotherapy in select low-risk early-stage triple-negative breast cancers^7,12^, evaluation of TILs presents further opportunities as a biomarker for radiation therapy. However, limited studies have evaluated the clinical relevance of TILs in the context of radiation in invasive breast cancer^13–15^. There is some evidence suggesting that high TILs may be prognostic for local recurrence and predictive of response to radiotherapy after breast conserving surgery and mastectomy, but results are conflicting^13,14^. The effect of TILs on regional nodal irradiation (RNI) is not known.

The benefit of RNI in addition to whole breast irradiation (WBI) after breast conserving surgery in patients with node-positive and high-risk node-negative disease was established in the Canadian Clinical Trial Group (CCTG) phase III MA.20 randomized controlled trial. Patients allocated to receive adjuvant RNI plus WBI experienced a significantly better disease-free survival (DFS) and distant disease-free survival (distant-DFS) compared to those who received WBI alone^16^.

The patient population of the MA.20 trial provided us with a unique opportunity to evaluate the impact of TILs on patient outcomes receiving radiotherapy. We hypothesized that baseline sTIL levels would provide prognostic information in early-stage node-positive and high-risk node-negative patients undergoing breast conserving surgery and WBI and potentially be predictive of the effect of additional RNI.

## Results

### Correlation of sTILs with clinicopathological features of the MA.20 translational study cohort

**Fig. 1:**
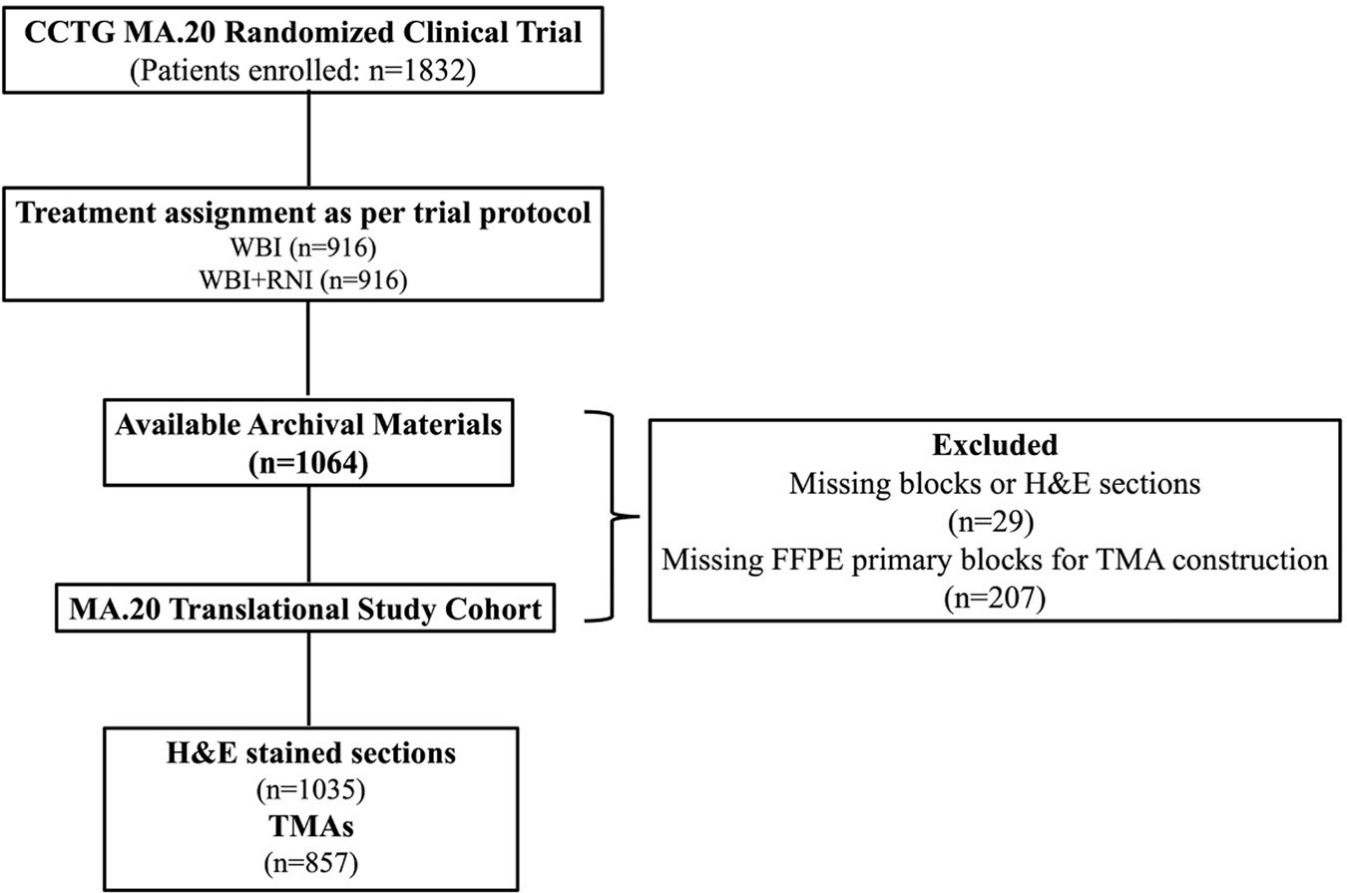
CONSORT diagram: the flow of participants from enrollment in the original MA.20 randomized clinical trial to the current biomarker-based translational study cohort. CCTG Canadian Clinical Trials Group, WBI whole breast radiation, RNI regional nodal radiation, FFPE formalin-fixed paraffin-embedded, TMA tissue microarray, H&E hematoxylin and eosin.

The CONSORT flow diagram of the study is presented in Fig. 1. Of the 1832 patients enrolled in the MA.20 clinical trial, 1064 cases were considered eligible for inclusion in the present translational study cohort based on the availability of the primary tumor blocks and/or the representative H&E-stained sections. A comparison between the original patient population of the MA.20 trial and the current translational study cohort is presented in Supplementary Table S2, showing that the baseline clinicopathological characteristics, treatments, and oncological outcomes were balanced between patients for whom biomarker values were or were not available. The mean age of the translational study cohort was 54 years. A total of 429 (49%) cases were categorized as luminal A and 440 (51%) as non-luminal A (luminal B = 221; HER2 positive = 48, basal = 154, TNBC = 17).

A total of 1035 sections were available for assessment of sTILs on H&E-stained sections. High levels of H&E sTILs (≥10%) were observed in 53% of tumors (mean = 17%, median = 10%, IQR, 5–20%) (Table 1 and Supplementary Fig. S2); these cases demonstrated a significant association with aggressive pathological features including tumor size >2 cm, grade III, axillary nodal positivity and hormone receptor-negative status (*P* < 0.05). As expected, there was a significant difference in the proportion of sTILs in the IHC-defined intrinsic subtypes, with 67% (*N* = 284/423) of luminal A breast cancers exhibiting low levels, whereas high sTIL were found in 69% (*N* = 294/429) of non-luminal A tumors (*P* < 0.0001) (Table 1).

**Table 1:** Correlation of sTILs assessed on H&E-stained sections and by CD8 immunohistochemistry with clinicopathological features in the full MA.20 translational study cohort

Clinicopathological parameters	H&E TILs		*P*-value	CD8 + sTILs		*P*-value
	<10%	≥10%		<16	≥16	
**Age (years)**			0.1			0.4
<50	156 (32)	199 (37)		139 (32)	149 (35)	
≥50	335 (68)	345 (63)		291 (68)	278 (65)	
**Tumor size (cm)**			0.0003			0.6
≤2	291 (59)	261 (48)		220 (51)	226 (53)	
>2	200 (41)	283 (52)		210 (49)	201 (47)	
**Tumor grade**			<0.0001			
I–II	344 (70)	234 (43)		254 (59)	217 (51)	0.01
III	143 (29)	307 (56)		172 (40)	208 (49)	
Unknown	4 (1)	3 (1)		4 (1)	2 (1)	
**ER status**			<0.0001			0.03
Negative	54 (11)	218 (40)		101 (24)	129 (30)	
Positive	437 (89)	326 (60)		329 (77)	298 (70)	
**PR status**			<0.0001			0.17
Negative	129 (26)	292 (54)		163 (38)	188 (44)	
Positive	360 (73)	251 (46)		265 (62)	238 (56)	
Missing	2 (0.4)	1 (0.2)		2 (1)	1 (0.2)	
**Nodal status**			0.01			0.1
Negative	29 (6)	59 (11)		48 (11)	31 (7)	
1–3 positive	429 (87)	458 (84)		361 (84)	368 (86)	
≥4 positive	33 (7)	27 (5)		21 (5)	28 (7)	
**IHC subtypes**			<0.0001			0.004
Non-luminal A	135 (28)	294 (54)		191 (44)	230 (54)	
Luminal A	284 (58)	139 (26)		227 (53)	184 (43)	
Missing	72 (15)	111 (20)		12 (3)	13 (3)	
**Radiation therapy assignment**			0.05			0.8
WBI	240 (49)	282 (52)		221 (51)	213 (50)	
WBI + RNI	246 (50)	262 (48)		207 (48)	213 (50)	
Not treated	5 (1)	0 (0)		2 (1)	1 (0.2)	

*sTILs* stromal tumor-infiltrating lymphocytes, *H&E* hematoxylin and eosin, *ER* estrogen receptor, *PR* progesterone receptor, *WBI* whole breast radiation, *RNI* regional nodal radiation, *IHC* immunohistochemistry.

Of the 857 cases evaluable on TMAs, CD8+ sTILs had a mean count per TMA core of 25 (median = 16, interquartile range, 7–35) (Table 1 and Supplementary Fig. S2). Luminal A tumors demonstrated a greater proportion of cases with CD8+ sTIL counts <16 (55%, *N* = 227/411). High levels of CD8+ sTILs showed a significant correlation with grade III tumors, ER negativity, and non-luminal A subgroup (*P* < 0.05) (Table 1).

### Prognostic association of sTILs in the full MA.20 translational study cohort

The prespecified primary objectives were evaluated in the full MA.20 translational study cohort. Follow-up was available for a median of 9.5 years from the time of randomization in the original clinical trial. In univariate analyses, no association was observed for H&E sTILs and CD8+sTILs for DFS either as a continuous or categorical variables. For secondary endpoints (distant-DFS and LR-DFS), no significant prognostic associations were observed for H&E sTILs. Increased CD8+ sTILs when analyzed as a continuous but not categorical variable was associated with an improvement in distant-DFS (HR 0.99, 95% CI 0.98–1.00, *P*-value 0.004) but not for LR-DFS (Supplementary Table S3).

**Fig. 2:**
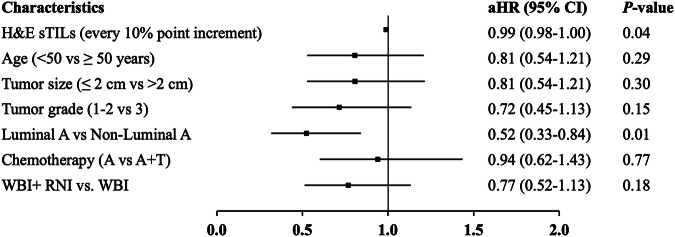
Multivariate Cox proportional analysis: forest plots showing adjusted HR (95% CI) for the prognostic value of H&E sTILs (continuous variable), clinicopathological and treatment-related factors for the secondary endpoint of distant-DFS in the full MA.20 translational study cohort (*N* = 736, events = 107). H&E, hematoxylin and eosin, WBI whole breast radiation, RNI regional nodal radiation, A anthracycline, T taxane, aHR adjusted hazard ratio, CI confidence interval, distant-DFS distant disease-free survival.

Multivariate analyses using Cox proportional hazard modeling were performed to investigate any independent prognostic value of sTILs after adjusting for clinicopathological (age, tumor size, grade, and breast cancer IHC subtypes) and treatment-related factors (chemotherapy and assignment to WBI with RNI versus WBI alone). No association was observed for H&E sTILs and DFS. Every 10% increment in H&E sTIL was associated with a significant improvement in distant-DFS (adjusted HR 0.99, 95% CI 0.98–1.0, *P* = 0.04) but not LR-DRS. (Fig. 2 and Supplementary Table 4). No associations were observed for H&E sTILs as a categorical variable (Supplementary Table 5).

On multivariate analyses the presence of CD8+ sTILs as a continuous variable was associated with a significant improvement in DFS (adjusted HR 0.99, 95% CI 0.98–1.00, *P* = 0.02) and remained a statistically relevant immune biomarker for distant-DFS, both when examined as a continuous (adjusted HR 0.98, 95% CI 0.97–0.99, *P* = 0.0002) and categorical variable (adjusted HR 0.66, 95% CI 0.44–0.98, *P* = 0.04). No significant associations were observed for CD8+ sTILs and LR-DFS (Tables 2 and 3).

**Table 2:** Multivariate Cox proportional analysis for the prognostic value of IHC assessed CD8+sTILs (categorical variable), clinicopathological and treatment-related factors for DFS, distant disease-free survival (distant-DFS), and locoregional disease-free survival (LR-DFS) in the MA.20 translational study cohort

Variables in the model	DFS (aHR, 95% CI)	*P*-value	Distant-DFS (aHR, 95% CI)	*P*-value	LR-DFS (aHR, 95% CI)	*P*-value
	*N* = *717, events* = *126*		*N* = *717, events* = *101*		*N* = *717, events* = *39*	
CD8+ sTILs (<16 vs. ≥16)	0.82 (0.58–1.17)	0.27	0.66 (0.44–0.98)	0.04	1.09 (0.57–2.06)	0.80
Age (<50 vs ≥50 years)	1.04 (0.73–1.49)	0.83	0.91 (0.60–1.36)	0.63	1.66 (0.88–3.13)	0.12
Tumor size (≤2 vs. >2 cm)	0.77 (0.53–1.13)	0.18	0.65 (0.42–0.99)	0.04	1.10 (0.54–2.22)	0.80
Tumor grade (1–2 vs. 3)	0.92 (0.61–1.40)	0.70	0.96 (0.61–1.53)	0.88	0.93 (0.43–2.00)	0.84
Non-luminal A vs. luminal A subtype	0.62 (0.41–0.95)	0.03	0.62 (0.39–0.99)	0.05	0.52 (0.24–1.15)	0.11
Chemotherapy (A vs. A+T)	0.91 (0.62–1.35)	0.65	0.96 (0.62–1.48)	0.86	0.64 (0.30–1.36)	0.24
Radiation (WBI vs WBI + RNI)	0.72 (0.50–1.02)	0.07	0.80 (0.54–1.19)	0.27	0.50 (0.26–0.97)	0.04

*H&E* hematoxylin and eosin, *A* anthracycline, *A+T* anthracycline and taxane, *WBI* whole breast radiation, *RNI* regional nodal radiation, *DFS* disease-free survival, *LR-DFS* locoregional disease-free survival, *CI* confidence interval, *aHR* adjusted hazard ratio.

**Table 3:** Multivariate Cox proportional analysis for the prognostic value of IHC assessed CD8+sTILs (continuous variable), clinicopathological and treatment-related factors for DFS, distant disease-free survival (distant-DFS), and locoregional disease-free survival (LR-DFS) in the MA.20 translational study cohort

Variables in the model	DFS (aHR, 95% CI)	*P*-value	Distant-DFS (aHR, 95% CI)	*P*-value	LR-DFS (aHR, 95% CI)	*P*-value
	*N* = *717, events* = *126*		*N* = *717, events* = *101*		*N* = *717, events* = *39*	
CD8+ sTILs	0.99 (0.98–1.00)	0.02	0.98 (0.97–0.99)	0.0002	1.00 (0.98–1.01)	0.56
Age (<50 vs ≥50 years)	1.03 (0.72–1.48)	0.86	0.90 (0.60–1.36)	0.62	1.66 (0.88–3.14)	0.12
Tumor size (≤2 vs. >2 cm)	0.79 (0.54–1.15)	0.22	0.66 (0.43–1.01)	0.06	1.11 (0.55–2.26)	0.77
Tumor grade (1–2 vs. 3)	0.90 (0.59–1.37)	0.61	0.93 (0.58–1.48)	0.75	0.90 (0.42–1.96)	0.80
Non-luminal A vs. luminal A subtype	0.59 (0.39–0.90)	0.02	0.58 (0.36–0.93)	0.02	0.50 (0.23–1.12)	0.09
Chemotherapy (A vs. A+T)	0.92 (0.62–1.36)	0.68	0.97 (0.63–1.49)	0.88	0.65 (0.30–1.38)	0.26
Radiation (WBI vs WBI + RNI)	0.71 (0.50–1.02)	0.06	0.80 (0.54–1.19)	0.27	0.50 (0.26–0.97)	0.04

*H&E* hematoxylin and eosin, *A* anthracycline, *A+T* anthracycline and taxane, *WBI* whole breast radiation, *RNI* regional nodal radiation, *DFS* disease-free survival, *LR-DFS* locoregional disease-free survival, *CI* confidence interval, *aHR* adjusted hazard ratio.

### The capacity of sTILs to predict benefit from regional nodal radiation in the full MA.20 translational study cohort

**Fig. 3:**
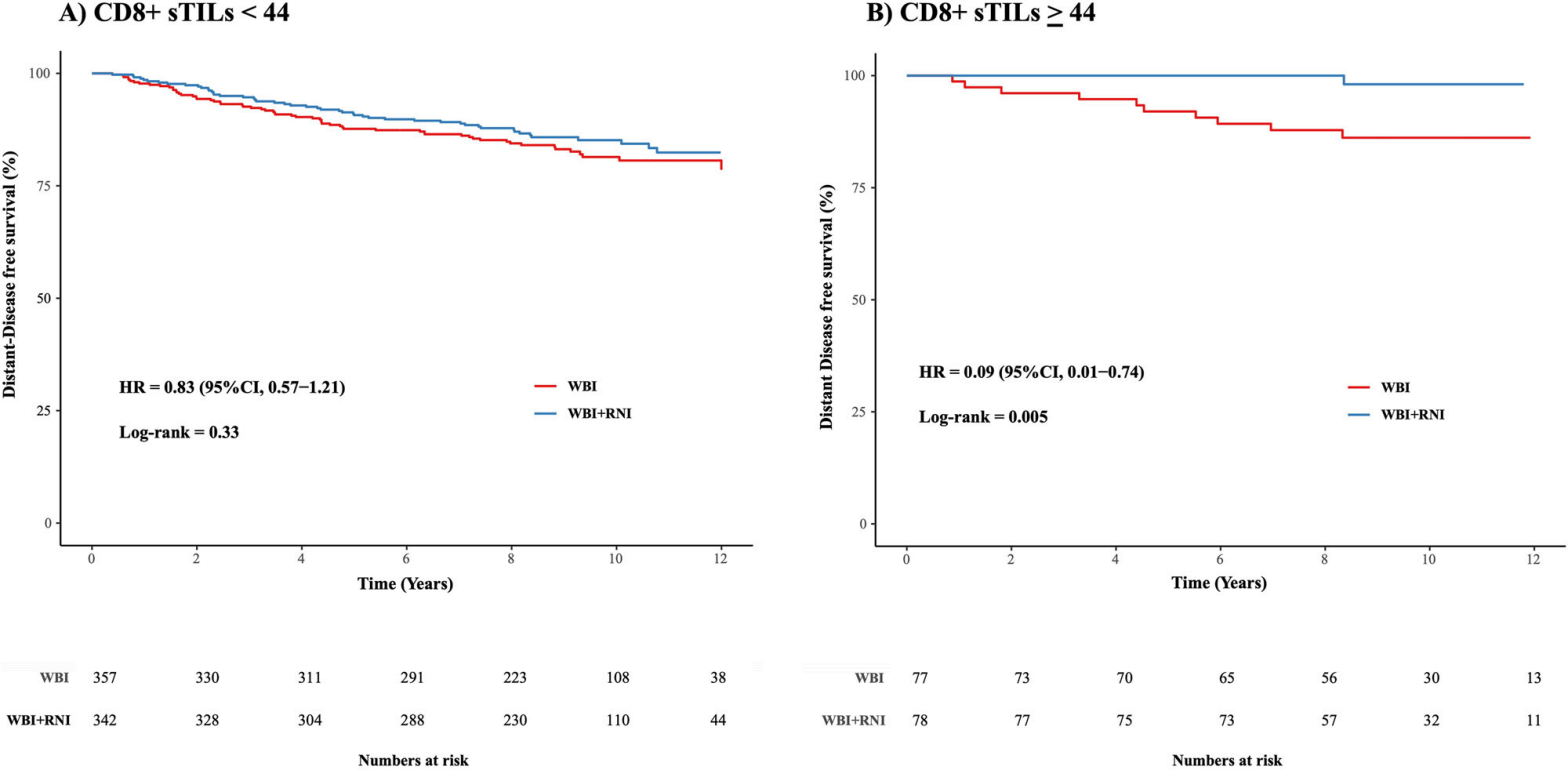
Survival probabilities for distant-DFS showing predictive value of CD8 sTILs. No difference in distant-DFS is observed in the WBI + RNI arm with low CD8 sTIL levels (<44) (**A**) versus an improved distant-DFS with high, ≥44 in the WBI + RNI arm (**B**). The *P*_(interaction)_ = 0.04. sTILs stromal tumor-infiltrating lymphocytes, WBI whole breast radiation, RNI regional nodal radiation, HR hazard ratio, distant-DFS distant disease-free survival.

Our co-primary objective was to investigate whether the effect of RNI plus WBI differed from WBI alone according to the extent of pre-treatment stromal lymphocytic infiltration in the MA.20 cohort. This was achieved by assessing the statistical significance of treatment by biomarker interaction. The results showed no significant interaction between RNI and sTILs for DFS for either assessed immune biomarkers at prespecified cutpoints (H&E sTILs: HR 1.20, 95% CI 0.75–1.91, *P*_(interaction)_ = 0.57) or (CD8+ sTILs: HR 0.07, 95% CI 0.41–1.23, *P*_(interaction)_ = 0.71). The predictive value of H&E sTILs also remained insignificant for distant-DFS (as categorical variable: HR 1.13, 95% CI 0.68–1.89, *P*_(interaction)_ = 0.56; as continuous variable: HR 0.98, 95% CI 0.86–1.13, *P*_(interaction)_ = 0.95) and LR-DFS (as categorical variable: HR 1.52, 95% CI 0.59–3.91, *P*_(interaction)_=0.80; continuous variable: HR 0.92, 95% CI 0.69–1.22, *P*_(interaction)_ = 0.29). CD8+ sTILs as a continuous variable but not as a pre-set categorical variable was associated with a statistically significant improvement in distant-DFS for patients randomized to WBI + RNI compared to those who received WBI alone (HR 0.98; 95%CI 0.96–1.00, *P*_(interaction)_ = 0.04). Utilizing a previously reported methodology, we performed an exploratory analysis for determining the optimal cutpoint for the predictive value of CD8 sTILs^17^. This showed that CD8+ sTILs provided a significant predictive value for improvement in distant-DFS for WBI + RNI compared to WBI alone at a cutpoint of 44 (CD8+ sTILs < 44, HR = 0.83; 95% CI 0.57–1.21), and (CD8+ sTILS ≥ 44; HR 0.09; 95% CI 0.01–0.74, *P*_(interaction)_ = 0.04) (Fig. 3). No other significant predictive associations were observed.

### Secondary objective: the prognostic and predictive capacity of sTILs in the IHC-defined luminal and non-luminal A subgroups

Given that luminal A breast cancers are mostly recognized for having low immunogenic potential compared to luminal B, basal (including TNBC), and HER2-positive breast cancer subtypes, prespecified subgroup analyses investigated the value of sTILs in IHC-defined luminal A versus non-luminal A for prognostication and predicting benefit from RNI. These analyses were performed for distant-DFS only, the endpoint for which statistically significant results were obtained for the primary objective. For prognostic analyses in non-luminal A subgroup, no associations were observed for H&E sTILs (as a continuous variable: HR 0.90, 95% CI 0.80–1.01, *P* = 0.08; as a categorical variable: HR 0.83, 95% CI 0.50–1.38, *P* = 0.48). CD8 sTILs analyzed both as continuous (HR 0.82, 95% CI 0.73–0.93, *P* = 0.001) and categorical variable (HR 0.55, 95% CI 0.34–0.91, *P* = 0.02) were associated with significantly improved distant-DFS independent of the standard clinicopathological variables in the non-luminal A subgroup. No prognostic associations were observed in the luminal A subgroup for either of the biomarkers (H&E sTILs as continuous variable: HR 0.83, 95% CI 0.58–1.20, *P* = 0.33; H&E sTILs as a categorical variable: HR 0.57, 95% CI 0.26–1.22, *P* = 0.15; CD8+sTILs as continuous variable: HR 0.83, 95% CI 0.67–1.03, *P* = 0.09; CD8+sTILs as categorical variable: HR 0.97, 95% CI 0.50–1.85, *P* = *0.92*). Neither of the immune biomarkers was predictive of a favorable benefit from RNI in the non-luminal A or luminal A subgroups at the predefined cutpoints nor as a continuous parameter (data not shown).

### Exploratory analyses

Although the optimum threshold for defining high versus low TILs is a work in progress and likely context-dependent, a cutoff of <30% versus ≥30% has shown the highest intraclass correlation coefficient and concordance among experienced pathologists in ring studies conducted by the International TIL Working Group^18^. No significant prognostic or predictive associations were observed using this cutpoint (data not shown). Lastly, our exploratory analyses examining the prognostic relevance of pre-treatment FOXP3+sTILs in the full translational study cohort did not achieve statistical significance for DFS and distant-DFS (Supplementary Table 6).

## Discussion

Our results demonstrated that H&E sTILs were not prognostic for DFS, but CD8+ sTILs, which identify cytotoxic T cells, were prognostic, suggesting that they may be more specific for an immune-activated state. Importantly, this effect was independent of age, tumor size, grade, breast cancer subtype, type of chemotherapy, and type of radiotherapy received. The positive results obtained by quantifying sTILs as a continuous parameter are not surprising, as a continuous expression of TILs has been reported to approximate in vivo immunological status and is the preferred method as per International TIL Working Group recommendations^19^.

On further examination, both H&E sTILs and CD8+ sTILs were prognostic for distant-DFS, the former as a continuous variable and the latter as both continuous and categorical variables. This effect was again independent of other prognostic variables and treatments. Further investigation indicated the prognostic effect was observed for non-luminal A cancers and not for luminal A type. These results are consistent with other studies in patients treated with adjuvant chemotherapy^3,6^.

Neither immune marker predicted the risk of LR-DFS. This may be because relatively few locoregional recurrences were observed in MA.20 compared to distant recurrences, as all patients were treated with WBI after breast conserving surgery. In a previous older Swedish trial in patients where the vast majority did not receive adjuvant systemic therapy, and were randomized to WBI or not after breast conserving surgery, H&E TILs were found to be prognostic for local recurrence, but the risk of local recurrence was much higher than observed in MA.20^13^.

Previous studies have indicated that high sTILs are predictive of pathological complete response in patients treated with neoadjuvant chemotherapy^6^. In our trial, H&E and CD8+ sTILs were not predictive for DFS, but CD8+ sTILs appeared to be predictive of greater improvement in patients treated with RNI plus WBI compared to WBI alone. These findings were based on an analysis of the secondary endpoint distant-DFS and were only positive for CD8+ sTILs. Nevertheless, the results are consistent with TILs evaluation in the Danish Breast Cancer Group postmastectomy trials^14^. In these trials, patients treated with adjuvant endocrine or chemotherapy were randomized to locoregional RT or not after mastectomy. High TILs were associated with a greater reduction in distant metastases in patients treated with locoregional RT. They too did not observe any effect of TILs on locoregional recurrence and suggested that the increased effect on distant recurrence may be mediated through a primed immune system activated by RT-induced cell death, leading to the release of tumor-associated neo-antigens. In MA.20 and the DBCG trials, distant metastases may in part be reduced by the sterilization of regional disease in the internal mammary or high axillary nodes that are not clinically evident but could lead to distant spread. The association of increased effects in patients with high TILs suggests that immune mechanisms may be additionally involved. With the current well-founded role of RNI in early breast cancer management^20,21^, the clinical importance of this finding remains unclear and merits further investigation. No predictive effect was demonstrated with sTILs assessed on H&E or by CD8 IHC for DFS or distant-DFS for the prespecified subgroup analyses comprising luminal A versus non-luminal A breast cancers, the latter of which have been demonstrated to be more immunogenic^14,22^. It is possible that the low number of events in the subgroups may have contributed to the observed lack of statistical significance.

Worth mentioning is our negative results for FOXP3+ sTILs. Regulatory T cells, a subset of CD4+T cells marked by FOXP3 expression, exhibit immunosuppressive functions. Their accumulation in the tumor microenvironment has been linked with poorer clinical outcomes and reduced effectiveness of immune checkpoint inhibitor therapies^23^. However, the prognostic significance of FOXP3+ TILs in breast cancer remains controversial. In our study, FOXP3+ sTILs did not show any significant prognostic value, which is consistent with other previous studies^22,24^. While technical variations may in part explain these results, emerging evidence suggests that FOXP3 expression represents a heterogeneous population of T cells, which includes both regulatory T cells and non-regulatory T cells influenced by a complex interplay of factors such as cellular metabolism^25^. Future studies incorporating multiplexing to identify functional subsets of FOXP3+ Tregs may inform prognosis and predictive relevance of T regulatory cells in the context of adjuvant radiation therapy in breast cancer.

The strengths of our study are that we were able to perform prognostic and predictive analyses for validated immune biomarkers in a formal retrospective-prospective, prespecified approach with independent execution of the statistical analyses. Our study has some limitations. Firstly, our predictive results for all case studies and for the predefined subgroup analyses may have been insignificant due to inadequate events and require validation in independent cohorts or related trials. Secondly, the inclusion of ER+ cases (from the luminal B subgroup) in the non-luminal A subgroup may have influenced the statistical inference for the prognostic and predictive significance of TILs in the HER2+ and basal/TNBC groups combined. Although overall ER+ breast cancers have demonstrated poor immunogenic potential, our rationale to perform subgroup analyses in luminal A versus non-luminal A subgroups (as opposed to luminal versus non-luminal subgroups) was based on differences in the immune profile of luminal A and B breast cancers, where the latter have shown higher TILs and prognostic relevance^26,27^. Thirdly, it is reasonable to consider that quantification of TILs on H&E-stained sections or by CD8 IHC may not capture all aspects of the tumor immune microenvironment, its stroma, and activation status^28–31^. Nevertheless, presently standardized TIL assessment on H&E slides is the recommended method for measuring tumor immunogenicity^21,32^. Lastly, while our cohort reflects the adjuvant treatment standards of the time, we acknowledge that systemic therapies have since evolved, including the broader adoption of taxanes, extended endocrine therapy, neoadjuvant approaches, and targeted therapies such as cdk4/6 inhibitors and immune checkpoint inhibitors. These therapies may alter the tumor immune microenvironment and affect TIL dynamics, potentially modifying their predictive value in contemporary settings.

Beyond the direct and indirect mechanisms of cellular cytotoxicity triggered by irreversible DNA damage, ionizing radiation can alter the properties of cancer cells and the associated microenvironment through complex immunobiological mechanisms that may boost host anti-tumoral immune^33^. Our study has shown the potential of sTILs for prognostication and prediction of the effects of RNI. The latter suggests that immune mechanisms may be responsible in part for the effectiveness of RNI and merits further investigation. Our findings may also help guide future clinical trials and translational research in several key areas. As interest grows in combining radiotherapy with immune checkpoint inhibitors, the immune contexture of the tumors, reflected in TIL composition, may serve as a biomarker for guiding patient selection and stratification, especially in the neoadjuvant setting^34–38^. Furthermore, having demonstrated the potential value of TILs in identifying TIL-enriched, low-risk subgroups of TNBC patients where chemotherapy omission may be considered^7^, similar approaches could be extended to other clinical scenarios. For instance, in the context of ongoing de-escalation strategies in axillary management (including omission of sentinel lymph node biopsy and radiation), immune contexture may contribute towards refining the clinical criteria for patient selection in clinical trials^39^.

## Methods

### Study population and prespecified endpoints

This translational study was conducted on pre-treatment archival tissue specimens of patients who participated in the MA.20 clinical trial, the study population of which has been described previously^16^. Briefly, MA.20 was a multicentre randomized clinical trial conducted between 2000-2007 and included 1832 women diagnosed with node-positive or high-risk node-negative invasive breast cancer treated with breast conserving surgery. Axillary management of the study participants included axillary dissection (level I–II) or a sentinel lymph node biopsy, which was converted to axillary dissection if the sentinel lymph node was positive for metastasis. The high-risk node-negative disease was defined as a tumor size of ≥5 cm, or ≥ 2 cm with either tumor grade 3, estrogen receptor (ER) negativity, or presence of lymphovascular invasion. All patients received adjuvant systemic therapy including chemotherapy (anthracycline with or without taxanes), endocrine (tamoxifen or aromatase inhibitors), and/or HER2 targeted therapies where indicated. Patients were randomized to receive either WBI alone (control arm, n = 916) or WBI in combination with ipsilateral RNI targeting the internal mammary, supraclavicular, and axillary regions (experimental arm, n = 916) as described previously^16^. Patients with T4 lesions, clinically fixed or matted axillary lymph nodes, metastatic involvement of internal mammary lymph nodes, or the presence of distant metastasis at diagnosis were considered ineligible for the trial.

Given in the original trial, the intervention arm experienced a significant improvement in DFS (defined as the time between randomization and the appearance of first local, regional, or distant recurrence or contralateral breast cancer), which was considered the primary endpoint of the current study. Distant disease-free survival, defined as the time between randomization and recurrence at a distant site or death due to breast cancer, and locoregional disease-free survival (LR-DFS, defined as the time between randomization and the first recurrence in the ipsilateral breast or axillary, supraclavicular, or internal mammary nodes without evidence of distant disease for 1 month) were the secondary endpoints.

### Prespecified primary, secondary, and exploratory objectives

With the prespecified endpoints for the full MA.20 translational study cohort, the primary objectives were to investigate the prognostic capacity of sTILs assessed on H&E-stained sections and by CD8 immunohistochemistry (IHC) as continuous and categorical parameters. The co-primary objective was to determine the capacity of the sTILs to predict the benefit of RNI. A secondary objective was to determine the prognostic and predictive importance of the immune biomarkers in IHC-defined luminal A versus non-luminal A subgroups for the endpoint/s that yielded statistically significant results for the primary objective. Other predefined exploratory analyses included an evaluation of the prognostic and predictive value of H&E sTILs using a <30% versus ≥30% cutpoint and prognostic value of FOXP3+ sTILs. An additional exploratory analysis (not prespecified) for the derivation of the optimal cutpoint for the predictive capacity of CD8 sTILs was also performed as described below.

### Description of biomarkers, laboratory procedures, and assessment criteria

Stromal TILs (assessed on H&E-stained sections) and their cytotoxic T-cell component (assessed by CD8 IHC on tissue microarrays) were considered the main biomarkers of adaptive anti-tumoral immune responses. FOXP3-expressing TILs, regulatory T-cell subsets involved in modulating immune evasion predominantly by inhibiting the effector functions of the cytotoxic T cells^40,41^, were considered as a secondary biomarker for the exploratory analyses.

Baseline sTILs were quantified on full-face H&E-stained sections prepared from the pre-treatment formalin-fixed paraffin-embedded tumor (FFPE) blocks according to the International TIL Working Group guidelines^42^. Briefly, sTILs were identified morphologically as mononuclear cells, inclusive of lymphocytes and plasma cells that exist within the tumor edge located in the stroma between the carcinoma cells without directly contacting or infiltrating tumor cell nests. Stomal TILs were reported as the percentage of the stromal area occupied by TILs over the total stromal area within the tumor, and the scores were recorded as a continuous variable^42^.

A total of 16 tissue microarrays (TMAs) with a core size of 0.6 mm were previously constructed (quadruplicate cores for each case) at CCTG, Queen’s University, Ontario, Canada, using the pre-treatment FFPE blocks prepared from surgically excised primary tumor tissues. These TMA blocks were sectioned at 4 μm on a microtome (Leica RM2235, Leica Biosystems). The IHC procedures for all the biomarkers utilized in this study were performed at the Molecular and Advanced Pathology Core Laboratory, Vancouver, BC, Canada, on a Bond Rx autostainer (Multiplex IHC Stainer Fully Automated-Leica Biosystems). Briefly, IHC was performed for CD8 and FOXP3 using previously validated assays^43,44^. Sections prepared from the TMAs were also stained for ER, progesterone receptor (PR), HER2, cytokeratin-5 (CK5) and epidermal growth factor receptor (EGFR) for assigning intrinsic subtypes based on standardized IHC assays as previously described: luminal A (ER and/or PR ≥ 1% / HER2 negative with PR > 20% and Ki67 < 14%), luminal B (ER and/ or PR ≥ 1% and PR < 20% or HER2 positive or Ki67 ≥ 14%), HER2 expressing (ER and PR negative and HER2 3+ or amplified), basal-like (ER/ PR and HER2 negative and CK5 and/or EGFR positive) and other TNBC (all markers negative)^45^. Antibodies and their respective IHC protocols are summarized in Supplementary Table S1.

Stained slides, including H&E sections, were digitally scanned on an Aperio AT2 (Leica Biosystems). Biomarkers were scored by pathologists (DG, AB, ZK, and ES) who were blinded to the clinicopathological and outcome data. For all IHC-stained biomarkers, a mean value was estimated for the replicates. Estrogen receptor, PR, HER2, Ki67, EGFR, and CK5 were scored as per the methods described earlier and utilized in the analyses as categorical variables^46^. For our primary objective, estimates of sTILs assessed on H&E sections and by CD8 IHC (absolute counts per TMA core) were recorded as continuous variables as reported previously^46^ and recoded into categorical variables by selecting the cutpoint determined by the median data value. Specifically, H&E sTILs were dichotomized at a previously validated cutpoint of <10% versus ≥10%^47^, which also approximated the median value of H&E TIL scores in the current study. The continuous scores for CD8 and FOXP3 sTILs were recoded into categories defined at the median score value, i.e., <16 versus ≥16 and <4 versus ≥4 per TMA core, respectively. For exploratory analyses, an additional cutpoint of 30% for sTILs was selected based on the literature^18,48^. To estimate an exploratory threshold of CD8+sTIL level that might define a subgroup with differential benefit from RNI, we applied a biomarker threshold model as described by Chen et al.^17^. This hierarchical approach treats the biomarker threshold value as a random variable, assigning it a probability distribution and using the observed data, both to estimate the threshold and to define a subgroup of patients with differential treatment benefit. This approach was applied to the continuously scored CD8+ sTIL variable, which was derived by averaging the scores across 4 TMA cores. As this threshold was data-driven and not prespecified in the study protocol, this analysis is considered exploratory. All biomarker scores were locked down before formal statistical analyses. Representative photomicrographs of the immune biomarkers are shown in Supplementary Fig. S1.

### Statistical analyses

Per ReMARK guidelines, the detailed strategy for analyses was preplanned and then shared with CCTG’s biostatistician (BC) in Kingston, Ontario, for independent execution. For descriptive statistics, Chi-square or Fisher’s exact test was applied to investigate the association between sTILs (H&E sTILs and CD8+sTILs) as categorical variables with clinicopathological factors, IHC subtypes and treatments. The prognostic significance of the biomarkers was determined by univariate analyses in the full cohort using the Kaplan-Meier methodology, and the survival estimates between the groups were compared by log-rank test. Multivariate Cox proportional hazard modeling was used to determine the independent prognostic significance of immune biomarkers, and adjusted hazard ratios (HR) with 95% confidence intervals (CI) were reported after applying the likelihood ratio test. Any deviations from the proportional hazard assumption were evaluated based on the Schoenfeld residuals. The following variables were adjusted in the multivariate model building: age at diagnosis (<50 vs. ≥50 years), tumor size (≤2 vs. >2 cm), tumor grade (1–2 vs. 3), subtype (luminal A vs. non-luminal A), type of chemotherapy (anthracycline vs. anthracycline + taxane), and treatment arm (WBI alone vs. WBI + RNI). Lymph node status was not included in the multivariate model due to limited variability within the cohort, with approximately 90% of patients being node positive.

For predictive analyses in the full cohort, we hypothesized that a significant interaction would exist between experimental treatment and sTIL status (continuous and categorical) and in prespecified subgroups of luminal A vs. non-luminal A. The interaction was tested by applying Cox regression modeling including sTILs (continuous and categorical), the experimental treatment, i.e, RNI, and the interaction term between the experimental treatment and sTILs. *P*-value of <0.05 was considered to be significant. All analyses were performed on SAS software (version 9.4, SAS Institute Inc., Cary, NC, USA).

### Ethics approval

The study was conducted in accordance with the ethical principles of the Declaration of Helsinki. All procedures were performed in compliance with relevant laws and institutional guidelines. Patients enrolled in the original MA.20 clinical trial provided written informed consent for the usage of their tumor specimens and anonymized clinicopathological data for relevant biomarker-based research approved by the Hamilton Integrated Research Ethics Board (HiREB Project # 3071; May 01, 2022). The experimental procedures performed in Vancouver were approved through a collaborative research agreement between McMaster University and the University of British Columbia.

## Supplementary information

Supplementary Data

## Data Availability

The data analyzed in this translational study are not publicly available because they contain individual patient information. The datasharing and access policy is detailed in https://www.ctg.queensu.ca/docs/public/policies/DataSharingandAccessPolicy.pdf.
