# Supplementary material for: Prognostic and predictive capacity of tumor infiltrating lymphocytes in the MA.20 regional node radiotherapy trial
Source: NPJ Breast Cancer. 2025 Aug 29;11:97. doi: 10.1038/s41523-025-00821-z (PMC12397239; doi:10.1038/s41523-025-00821-z)
Supplement: Supplementary file 1 — Supplementary Data [file 41523_2025_821_MOESM1_ESM.docx]

**Supplementary Data File:**

**Supplementary Table S1 |** Details of the antibodies and protocols for immunohistochemistry

| **Biomarker** | **Antibody** | **Catalogue ID** | **Antigen retrieval*** | **Dilution** | **Incubation time (primary antibody)** | **Detection system / Secondary antibody (incubation time)** | **Incubation time with hematoxylin** |
| --- | --- | --- | --- | --- | --- | --- | --- |
| ER | Invitrogen | MA5-14501 | ER2-20 min | 1:200 | 30 min | BOND Polymer Refine Detection / Polymer (part of detection kit) 8min | 6 min |
| PR | Dako | M356801-2 | ER1-20 min | 1:3000 | 30 min |  |  |
| HER2 | Thermo Scientific | RM-9103-S0 | ER1-20 min | 1:100 | 15min |  |  |
| Ki67 | Dako | M724029-2 | ER2-20 min | 1:100 | 30min |  |  |
| EGFR | Epitomics | AC-0025 | ER-20 min | 1:100 | 30 min |  |  |
| CK5 | Invitrogen | MA5-12596 | ER1-20 min | 1:100 | 30 min |  |  |
| CD8 | Dako | M710301-2 | ER1-20min | 1:100 | 15 min |  |  |
| FOXP3 | Abcam | ab20034 | ER1-20min | 1:200 | 30 min |  |  |

**Abbreviations:** ER, estrogen receptor; PR, progesterone receptor; HER2, human epidermal growth factor receptor-2; EGFR, epidermal growth factor receptor; CK5, cytokeratin 5; FOXP3, forkhead box protein P3; CD8, cluster of differentiation 8.

*ERI, BOND Epitope Retrieval Solution 1 (citrate-based, pH 6); ER2, BOND Epitope Retrieval Solution 2 [EDTA (Ethylenediaminetetraacetic acid based, pH 9)].


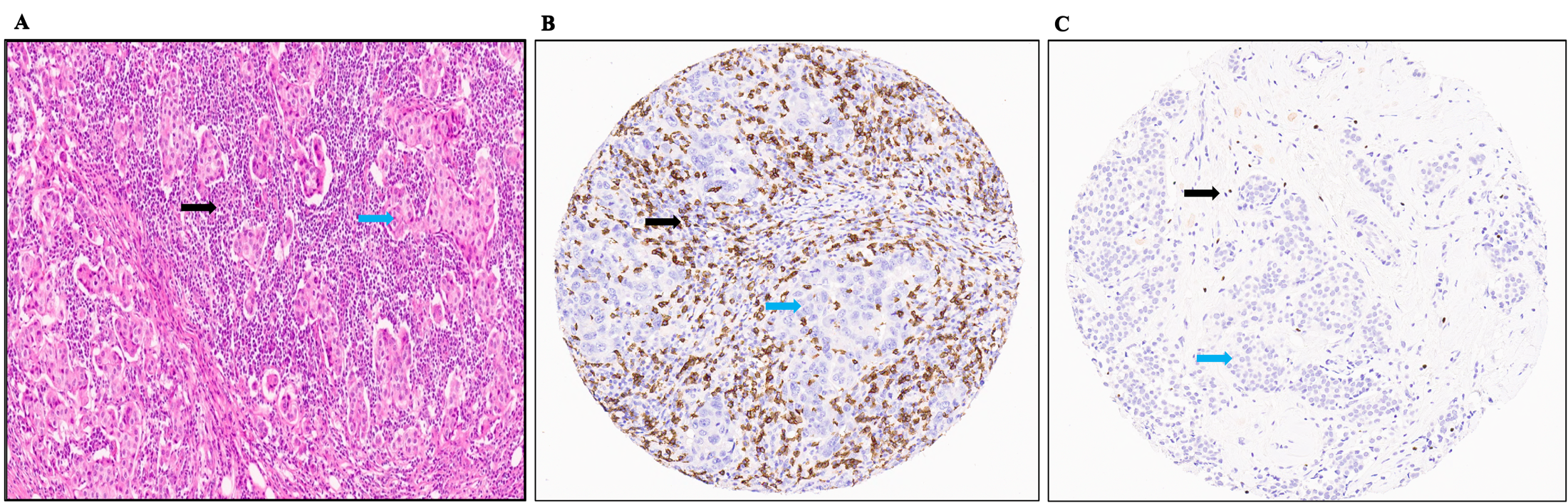


**Supplementary Figure S1|** Representative photomicrographs of the immune biomarkers included in the MA.20 translational study: (A) high density of sTILs (≥10%) on a full-face H&E stained breast cancer section; (B) high density of CD8+ sTILs (≥16) on a TMA section; (C) FOXP3+ sTILs (≥4) on a TMA section (black arrows indicate the immune cells and blue arrows indicate breast carcinoma cells). The images were acquired at 10X original objective lens magnification.

**Abbreviations:** sTILs, stromal tumor infiltrating lymphocytes; TMA, tissue microarray; H&E, hematoxylin and eosin; CD8, cluster of differentiation 8; FOXP3, forkhead box protein P3.

**Supplementary Table S2|** Comparison of the baseline clinicopathological characteristics and oncological outcomes of the patients with and without the biomarker values from the original MA.20 clinical trial.

| **Characteristics** | **Patients with**  **biomarker values**  ***N* (%)** | **Patients without**  **biomarker values**  ***N* (%)** | **Total**  ***N* (%)** | ***P-*value** |
| --- | --- | --- | --- | --- |
| **Age** |  |  |  | 0.9 |
| <50 | 365 (34) | 265 (35) | 630 (34) |  |
| ≥50 | 699 (66) | 503 (66) | 1202 (66) |  |
|  |  |  |  |  |
| **Tumor size** |  |  |  | 0.2 |
| ≤ 2cm | 570 (54) | 390 (51) | 960 (52) |  |
| > 2cm | 494 (46) | 377 (49) | 871 (48) |  |
| Missing | 0 (0) | 1 (0.1) | 1 (0.1) |  |
|  |  |  |  |  |
| **SBR Tumor grade** |  |  |  | 0.4 |
| I-II | 596 (56) | 444 (58) | 1040 (57) |  |
| III | 461 (43) | 317 (41) | 778 (43) |  |
| Unknown | 7 (1) | 7 (1) | 14 (1) |  |
|  |  |  |  |  |
| **ER status** |  |  |  | 0.2 |
| Negative | 281 (26) | 184 (24) | 465 (25) |  |
| Positive | 783 (74) | 584 (76) | 1367 (75) |  |
|  |  |  |  |  |
| **PR status** |  |  |  | 0.5 |
| Negative | 433 (41) | 292 (38) | 725 (40) |  |
| Positive | 628 (59) | 474 (62) | 1102 (60) |  |
| Missing | 3 (0.3) | 2 (0.3) | 5 (0.3) |  |
|  |  |  |  |  |
| **HER2 neu testing** |  |  |  |  |
| No | 400 (38) | 259 (34) | 659 (36) | N/A |
| Yes | 664 (62) | 509 (66) | 1173 (64) |  |
|  |  |  |  |  |
| **HER2 neu status** |  |  |  | 0.1 |
| 0 or 1+ | 532 (50) | 415 (54) | 947 (52) |  |
| 2+ | 39 (4) | 35 (5) | 74 (4) |  |
| 3+ | 85 (8) | 54 (7) | 139 (8) |  |
| Missing | 4 (0.4) | 5 (1) | 9 (1) |  |
| Unknown | 4 (0.4) | 0 (0) | 4 (0.2) |  |
| Not applicable | 400 (38) | 259 (34) | 659 (36) |  |
|  |  |  |  |  |
| **Number of positive nodes** |  |  |  | 0.06 |
| None | 90 (9) | 87 (11) | 177 (10) |  |
| 1-3 | 911 (86) | 647 (84) | 1558 (85) |  |
| 4+ | 63 (6) | 34 (4) | 97 (5) |  |
|  |  |  |  |  |
| **Adjuvant chemotherapy** |  |  |  | 0.002 |
| Other | 37 (4) | 56 (7) | 93 (5) |  |
| Anthracycline without taxane | 637 (60) | 457 (60) | 1094 (60) |  |
| Anthracycline with taxane | 288 (27) | 185 (24) | 473 (26) |  |
| None | 102 (10) | 70 (10) | 172 (9) |  |
|  |  |  |  |  |
| **Adjuvant endocrine therapy** |  |  |  | 0.6 |
| Any AI | 114 (11) | 78 (10) | 192 (11) |  |
| Tamoxifen only | 483 (45) | 331 (43) | 814 (44) |  |
| None | 463 (44) | 354 (46) | 817 (45) |  |
| Unknown | 4 (0.4) | 5 (1) | 9 (1) |  |
|  |  |  |  |  |
| **Treatment arm** |  |  |  | 0.5 |
| WBI | 543 (51) | 384 (50) | 927 (51) |  |
| WBI+RNI | 516 (49) | 377 (49) | 893 (49) |  |
| Not treated | 5 (1) | 7 (1) | 12 (1) |  |
|  |  |  |  |  |
| **Patients who died** | 179 (17) | 144 (19) | 323 (18) | N/A |
| Cause of death | | | | |
| Disease | 114 (11) | 90 (12) | 204 (11) |  |
| Others | 53 (5) | 49 (6) | 102 (6) |  |
| Unknown | 12 (1) | 5 (1) | 17 (1) |  |
|  |  |  |  |  |
| **Type of recurrences** |  |  |  | N/A |
| Local or regional recurrence | 54 (5) | 47 (6) | 101 (6) |  |
| Local recurrence only | 38 (4) | 33 (4) | 71 (4) |  |
| Regional recurrence only | 14 (1) | 14 (2) | 28 (2) |  |
| Local and regional recurrence | 2 (0.2) | 0 (0) | 2 (0.1) |  |
| Distant Recurrence | 151 (14) | 118 (15) | 269 (15) |  |
| Local or regional recurrence within 30 days of distant recurrence | 9 (1) | 7 (1) | 16 (1) |  |
| Distant recurrence after local or regional recurrence | 32 (3) | 19 (3) | 51 (3) |  |
| Death with/from breast cancer without distant recurrence | 2 (0.2) | 1 (0.1) | 3 (0.2) |  |
| Distant recurrence only | 106 (10) | 84 (11) | 190 (10) |  |
| Distant recurrence before local or regional recurrence | 2 (0.2) | 7 (1) | 9 (1) |  |

**Abbreviations:** WBI, whole breast radiation; RNI, regional nodal radiation; SBR, Scarff- Bloom Richardson; ER, estrogen receptor; PR, progesterone receptor; HER2neu, human epidermal growth factor receptor 2; AI, aromatase inhibitors.


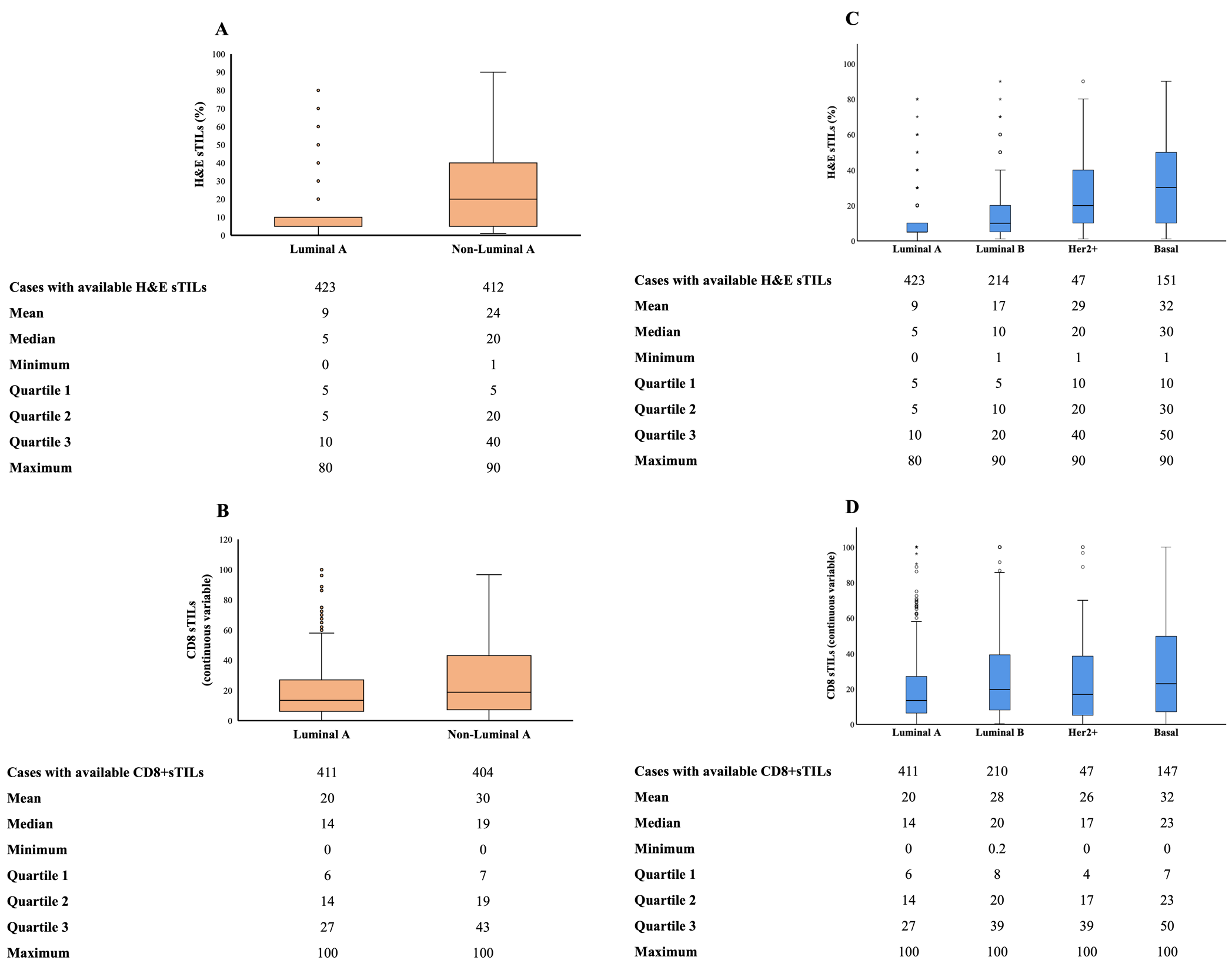


**Supplementary Figure S2 |** Box plots showing the descriptive statistics for H&E sTILs (A&C) and CD8+sTILs (B&D) within the breast cancer IHC-defined breast cancer subtypes.

**Abbreviations:** H&E, hematoxylin and eosin; CD8, cluster of differentiation 8; sTILs, stromal tumor infiltrating lymphocytes; HER2, human epidermal growth factor receptor 2; IHC, immunohistochemistry.

**Supplementary Table S3|** Univariate prognostic significance of sTILs assessed on H&E stained sections and by immunohistochemistry of CD8 in the full MA.20 translational study cohort for the primary endpoint of disease free survival (DFS) and secondary endpoints of distant disease free survival (distant-DFS) and locoregional disease free survival (LR-DFS). The biomarkers were assessed as continuous and categorical parameters (H&E sTILs: low, <10% versus high, ≥10%; CD8+ sTILs, low, <16 versus high, ≥16).

| **Immune biomarkers** | **Patients**  **(*N*)** | **DFS** | | | **Distant-DFS** | | | **LR-DFS** | | |
| --- | --- | --- | --- | --- | --- | --- | --- | --- | --- | --- |
|  |  | **HR (95% CI)** | ***P*-value** | **Events** | **HR (95% CI)** | ***P*-value** | **Events** | **HR (95% CI)** | ***P*-value** | **Events** |
| **sTILs assessed on H&E sections** | | | | | | | | | | |
| Continuous parameter | 1035 | 1.002 (1.00-1.01) | 0.61 | 184 | 1.00 (0.99-1.01) | 0.69 | 147 | 1.01 (0.99-1.02) | 0.44 | 53 |
| Categorical parameter |  | 1.09 (0.82-1.46) | 0.56 |  | 1.01 (0.73-1.39) | 0.97 |  | 1.42 (0.82-2.46) | 0.21 |  |
| **CD8+sTILs assessed by IHC** | | | | | | | | | | |
| Continuous parameter | 857 | 0.99 (0.99-1.001) | 0.09 | 153 | 0.99 (0.98-1.00) | 0.004 | 123 | 1.00 (0.99-1.01) | 0.98 | 42 |
| Categorical parameter |  | 0.90 (0.66-1.24) | 0.53 |  | 0.77 (0.54-1.10) | 0.16 |  | 1.17 (0.64-2.16) | 0.60 |  |

**Abbreviations:** sTILs, stromal tumor infiltrating lymphocytes; CD8, cluster of differentiation 8; H&E, hematoxylin and eosin; HR, hazard ratio; CI, confidence interval; IHC, immunohistochemistry; DFS, disease free survival; distant-DFS, distant disease free survival; LR-DFS, locoregional disease free survival

**Supplementary Table S4 |** Multivariate Cox proportional analysis for the prognostic value of H&E sTILs (continuous variable), clinicopathological and treatment related factors for disease free survival (DFS), distant disease free survival (distant-DFS) and locoregional disease free survival (LR-DFS) in the MA.20 translational study cohort

| **Variables in the model** | **DFS (aHR, 95% CI)** | ***P*-value** | **Distant-DFS**  **(aHR, 95% CI)** | ***P*-value** | **LR-DFS**  **(aHR, 95% CI)** | ***P*-value** |
| --- | --- | --- | --- | --- | --- | --- |
|  | *N=736, events 133* | | *N=736, events 107* | | *N=736, events=41* | |
| H&E sTILs (each 10% point increase) | 0.99 (0.98-1.00) | 0.18 | 0.99 (0.98-1.00) | 0.04 | 0.99 (0.97- 1.01) | 0.30 |
| Age (<50 vs ≥50 years) | 0.97 (0.68- 1.38) | 0.85 | 0.81 (0.54- 1.21) | 0.29 | 1.66 (0.89- 3.08) | 0.11 |
| Tumor size (≤ 2 vs. >2 cm) | 0.89 (0.62- 1.29) | 0.55 | 0.81 (0.54- 1.21) | 0.30 | 0.99 (0.50-1.96) | 0.97 |
| Tumor grade (1-2 vs. 3) | 0.75 (0.49- 1.13) | 0.17 | 0.72 (0.45- 1.13) | 0.15 | 0.86 (0.40- 1.85) | 0.70 |
| Non-luminal A vs. luminal A subtype | 0.56 (0.36- 0.85) | 0.01 | 0.52 (0.33- 0.84) | 0.007 | 0.49 (0.23- 1.06) | 0.07 |
| Chemotherapy (A vs. A+T) | 0.89 (0.61- 1.31) | 0.56 | 0.94 (0.62- 1.43) | 0.77 | 0.60 (0.28- 1.26) | 0.18 |
| Radiation (WBI vs WBI+RNI) | 0.72 (0.51- 1.01) | 0.06 | 0.77 (0.52- 1.13) | 0.18 | 0.56 (0.29- 1.05) | 0.07 |

**Abbreviations:** H&E, hematoxylin and eosin; A, anthracycline; A+T, anthracycline and taxane; WBI, whole breast radiation; RNI, regional nodal radiation; DFS, disease-free survival; distant-DFS, distant disease free survival; LR-DFS, locoregional disease-free survival; CI, confidence interval; aHR, adjusted hazard ratio

**Supplementary Table S5 |** Multivariate Cox proportional analysis for the prognostic value of H&E sTILs (categorical variable), clinicopathological and treatment related factors for the primary and secondary endpoints in the MA.20 translational study cohort

| **Variables in the model** | **DFS (aHR, 95% CI)** | ***P*-value** | **Distant-DFS**  **(aHR, 95% CI)** | ***P*-value** | **LR-DFS**  **(aHR, 95% CI)** | ***P*-value** |
| --- | --- | --- | --- | --- | --- | --- |
|  | *N= 736, events=133* | | *N=736, events=107* | | *N=736, events=41* | |
| H&E sTILs (<10% vs. ≥10%) | 0.80 (0.55-1.16) | 0.24 | 0.72 (0.48-1.09) | 0.12 | 0.84 (0.43-1.64) | 0.62 |
| Age (<50 vs ≥50 years) | 0.96 (0.68-1.37) | 0.83 | 0.80 (0.53-1.19) | 0.27 | 1.65 (0.89-3.07) | 0.11 |
| Tumor size (≤ 2 vs. >2 cm) | 0.88 (0.61-1.26) | 0.48 | 0.79 (0.52-1.18) | 0.24 | 0.96 (0.49-1.92) | 0.92 |
| Tumor grade (1-2 vs. 3) | 0.77 (0.51-1.17) | 0.22 | 0.76 (0.48-1.20) | 0.25 | 0.92 (0.43-1.96) | 0.83 |
| Non-luminal A vs. luminal A subtype | 0.56 (0.37-0.86) | 0.01 | 0.54 (0.33-0.86) | 0.01 | 0.51 (0.24-1.12) | 0.09 |
| Chemotherapy (A vs. A+T) | 0.89 (0.61-1.30) | 0.55 | 0.94 (0.62-1.44) | 0.79 | 0.60 (0.28-1.26) | 0.18 |
| Radiation (WBI vs WBI+RNI) | 0.72 (0.51-1.02) | 0.06 | 0.78 (0.53-1.15) | 0.21 | 0.56 (0.30-1.06) | 0.08 |

**Abbreviations:** H&E, hematoxylin and eosin; A, anthracycline; A+T, anthracycline and taxane; WBI, whole breast radiation; RNI, regional nodal radiation; DFS, disease free survival; LR-DFS, locoregional disease-free survival; CI, confidence interval; aHR, adjusted hazard ratio.

**Supplementary Table S6 |** Univariate prognostic significance of FOXP3+sTILs in the full MA.20 translational study cohort for the primary endpoint of disease free survival (DFS) and secondary endpoints of distant disease free survival (distant-DFS) and locoregional disease free survival (LR-DFS). The biomarker was assessed as a continuous and categorical parameter (scores dichotomized as low, <4 versus high, ≥4).

| **Immune biomarker** | **Patients**  **(*N*)** | **DFS** | | | **Distant-DFS** | | | **LR-DFS** | | |
| --- | --- | --- | --- | --- | --- | --- | --- | --- | --- | --- |
|  |  | **HR (95% CI)** | ***P*-value** | **Events** | **HR (95% CI)** | ***P*-value** | **Events** | **HR (95% CI)** | ***P*-value** | **Events** |
| **FOXP3+ sTILs** | | | | | | | | | | |
| Continuous parameter | 862 | 1.00 (0.98-1.01) | 0.53 | 159 | 0.99 (0.98-1.01) | 0.45 | 130 | 0.99 (0.97-1.02) | 0.61 | 43 |
| Categorical parameter |  | 0.88 (0.65- 1.21) | 0.43 |  | 0.84 (0.59-1.18) | 0.31 |  | 0.95 (0.52-1.73) | 0.87 |  |

**Abbreviations:** sTILs, stromal tumor infiltrating lymphocytes; HR,hazard ratio; CI, confidence interval; DFS, disease free survival; distant-DFS, distant disease free survival; LR-DFS, locoregional disease free survival.
